# 799. Unstable Housing, Daily Drug Use, and Transactional Sex are Associated with Viremia in Transgender Individuals Living with HIV

**DOI:** 10.1093/ofid/ofac492.059

**Published:** 2022-12-15

**Authors:** Grace Garrett, Rahwa Eyasu, Amelia A Cover, Ashley Davis, Omar Harfouch, Emade Ebah, Phyllis Bijole, Catherine Gannon, Vivian Wang, Michelle Spikes, F N U Imani, Miriam Jones, Henry Masur, Shyam Kottilil, Sarah Kattakuzhy, Elana S Rosenthal

**Affiliations:** Critical Care Medicine Department, National Institutes of Health (NIH), Washington, DC; Institute for Human Virology (IHV), University of Maryland School of Medicine, Washington, District of Columbia; Institute for Human Virology (IHV), University of Maryland School of Medicine, Washington, District of Columbia; Institute for Human Virology (IHV), University of Maryland School of Medicine, Washington, District of Columbia; University of Maryland – School of Medicine. Department of Infectious Diseases., Baltimore, Maryland; Institute for Human Virology (IHV), University of Maryland School of Medicine, Washington, District of Columbia; HIPS.org, Washington, District of Columbia; Critical Care Medicine Department, National Institutes of Health (NIH), Washington, DC; Critical Care Medicine Department, National Institutes of Health (NIH), Washington, DC; HIPS.org, Washington, District of Columbia; HIPS.org, Washington, District of Columbia; HIPS.org, Washington, District of Columbia; Critical Care Medicine Department, National Institutes of Health (NIH), Washington, DC; Institute for Human Virology (IHV), University of Maryland School of Medicine, Washington, District of Columbia; Institute for Human Virology (IHV), University of Maryland School of Medicine, Washington, District of Columbia; Institute for Human Virology (IHV), University of Maryland School of Medicine, Washington, District of Columbia

## Abstract

**Background:**

In the United States, HIV is highly prevalent among transgender individuals, but factors contributing to viremia in this population are poorly understood. We aimed to assess factors associated with HIV viremia among transgender people living with HIV (PLWH).

**Methods:**

PATCH is a longitudinal natural history study of transgender individuals in Washington, DC. Participants complete laboratory assessments, as well as surveys assessing demographics, behaviors, and mental health. Patients whose HIV viral load was >200 copies/mL at screening were considered viremic. Fisher’s exact test was used for statistical analysis.

**Results:**

Of 62 enrolled participants, 35(56%) were HIV+, the majority of whom were prescribed antiretroviral therapy (31, 89%) and had an undetectable viral load (25, 71%). Of PLWH, 35(100%) were assigned male at birth, 34(97%) were Black, and the median age was 37 years. PLWH were predominately stably housed (24, 69%), with 9(26%) reporting daily drug use or more, and 16(46%) reporting transactional sex within a year.

When comparing PLWH who were viremic versus those virally suppressed, viremic PLWH were less likely to be prescribed antiretroviral therapy (60% vs 100%, p=0.004), and more likely to have unstable housing (60% vs 25%, p=0.04), use drugs daily or more in the last month (60% vs 12%, p=0.007), and engage in transactional sex in the last 12 months (80% vs 32%, p=0.02). HIV viral suppression was not significantly associated with number of sexual partners within the last 12 months or always using condoms during any type of sex (p >0.05).

**Conclusion:**

In this cohort of transgender PLWH, we found moderate rates of HIV viremia associated with chaotic life factors such as daily drug use, unstable housing, and engaging in transactional sex. In addition, we found low rates of consistent condom use despite multiple sexual partners, regardless of viral load. Comprehensive care programs that improve access to ART, address substance use disorders and facilitate housing may serve to improve treatment outcomes and reduce risk of HIV transmission in this population.

**Disclosures:**

**Shyam Kottilil, MD, PhD**, Arbutus Pharmaceuticals: Grant/Research Support|Gilead: Grant/Research Support|Merck: Grant/Research Support|Regeneron Pharmaceuticals: Advisor/Consultant|Silverback Therapeutics: Advisor/Consultant|The Liver Company: Advisor/Consultant|Yufan Biotechnologies: Advisor/Consultant **Elana S. Rosenthal, MD**, Gilead Sciences: Grant/Research Support|Merck: Grant/Research Support.

**Table 1:**
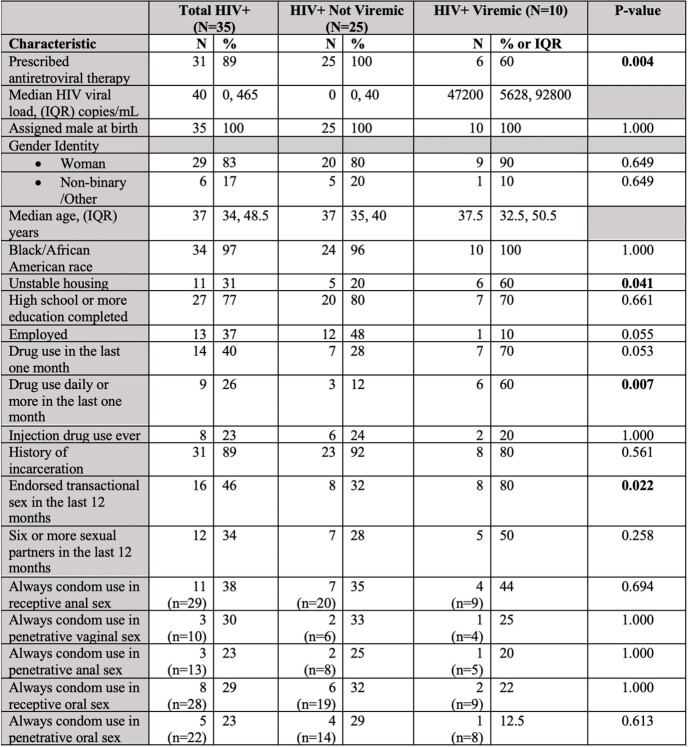
Characteristics of Transgender Individuals with HIV by Viremic Status

